# Histological evaluation of PAXgene tissue fixation in Barrett’s esophagus and esophageal adenocarcinoma diagnostics

**DOI:** 10.1007/s00428-022-03471-9

**Published:** 2022-12-17

**Authors:** Melissa Barroux, Julia Horstmann, Lisa Fricke, Linus Schömig, Martin Werner, Ekaterina Kraynova, Katerina Kamarádová, Jean-François Fléjou, Bruno Maerkel, M. Priyanthi Kumarasinghe, Michael Vieth, Maria Westerhoff, Deepa T. Patil, Katja Steiger, Karl-Friedrich Becker, Wilko Weichert, Roland M. Schmid, Michael Quante, Julia Slotta-Huspenina

**Affiliations:** 1 Klinikum Rechts Der Isar, Medical Clinic and Polyclinic II, Technical University of Munich, Munich, Germany; 2 Department of Medicine II, Universitaetsklinikum Freiburg, Freiburg, Germany; 3 Institute for Surgical Pathology, Medical Center-University of Freiburg and Faculty of Medicine, University of Freiburg, 79106 Freiburg, Germany; 4 Department of Pathology, Yaroslavl Regional Cancer Hospital, Yaroslavl, Russian Federation; 5 The Fingerland Department of Pathology, Faculty of Medicine and University Hospital, Charles University, Hradec Králové, Czech Republic; 6 Service d’Anatomie Pathologique, AP-HP, Faculté de Médecine Sorbonne, Hôpital Saint-Antoine, Université, 75012 Paris, France; 7 Institute of Pathology and Molecular Diagnostics, University Medical Center Augsburg, Augsburg, Germany; 8 Department of Pathology, PathWest Laboratory-University of Western Australia, WA Perth, Australia; 9 Institute for Pathology, Friedrich-Alexander-University Erlangen-Nuremberg, Klinikum Bayreuth, Bayreuth, Germany; 10 Department of Pathology, Michigan Medicine, Ann Arbor, MI USA; 11 Department of Pathology, Brigham and Women’s Hospital and Harvard Medical School, Boston, USA; 12 Institute of Pathology, Technical University of Munich, Munich, Germany

**Keywords:** Esophageal adenocarcinoma, Dysplasia, PAXgene-fixed paraffin-embedded

## Abstract

The dysplasia grading of Barrett’s esophagus (BE), based on the histomorphological assessment of formalin-fixed, paraffin-embedded (FFPE) tissue, suffers from high interobserver variability leading to an unsatisfactory prediction of cancer risk. Thus, pre-analytic preservation of biological molecules, which could improve risk prediction in BE enabling molecular and genetic analysis, is needed. We aimed to evaluate such a molecular pre-analytic fixation tool, PAXgene-fixed paraffin-embedded (PFPE) biopsies, and their suitability for histomorphological BE diagnostics in comparison to FFPE. In a ring trial, 9 GI pathologists evaluated 116 digital BE slides of non-dysplastic BE (NDBE), low-grade dysplasia (LGD), high-grade dysplasia (HGD), and esophageal adenocarcinomas (EAC) using virtual microscopy. Overall quality, cytological and histomorphological parameters, dysplasia criteria, and diagnosis were analyzed. PFPE showed better preservation of nuclear details as chromatin and nucleoli, whereas overall quality and histomorphologic parameters as visibility of basal lamina, goblet cells, and presence of artifacts were scored as equal to FFPE. The interobserver reproducibility with regard to the diagnosis was best for NDBE and EAC (*κ*_*F*_ = 0.72–0.75) and poor for LGD and HGD (*κ*_*F*_ = 0.13–0.3) in both. In conclusion, our data suggest that PFPE allows equally confident histomorphological diagnosis of BE and EAC, introducing a novel tool for molecular analysis and parallel histomorphological evaluation.

## Introduction

Barrett’s esophagus (BE) is a premalignant condition which predisposes to esophageal adenocarcinomas (EAC). BE is defined histopathologically as the replacement of stratified squamous epithelium of the distal esophagus by columnar epithelium that can contain intestinal metaplasia. Routine histomorphological assessment of BE and grading of dysplasia from formalin-fixed, paraffin-embedded (FFPE) biopsies remains the gold standard for risk stratification for patients according to their perceived progression risk to EAC [1–3]. The Vienna classification for grading of BE categorizes lesions as negative for dysplasia (NDBE), indefinite for dysplasia (IFD), low-grade dysplasia (LGD), high-grade dysplasia (HGD), and invasive neoplasia based on architectural and cytological features [2]. These include gland architecture, loss of surface maturation, and cytological abnormalities such as enlarged nuclei or any size variability of nuclei and mitosis. Although the Vienna classification has improved the international diagnostic classification of gastrointestinal epithelial neoplastic lesions, the agreement of dysplasia grading is moderate to poor due to substantial interobserver variability (highest among LGD *κ* = 0.11–0.31) [4–6], even among expert pathologists. Moreover, the prediction of cancer risk from histomorphological grading on BE remains limited due to the fact that dysplasia does not reliably predict progression to cancer, and non-dysplastic BE does not provide any morphological features that could be used for risk assessment [7, 8]. Notably, abnormal p53 immunohistochemistry (IHC) is strongly associated with BE at higher risk of progression, including patients without evidence of dysplasia [9]. Recent studies showed that molecular markers such as changes in copy number patterns and the degree of clonal diversity are promising biomarkers in BE and could predict cancer progression years ahead [7, 10–14]. These findings indicate a high potential of objective (molecular) biomarkers for future clinical practice and the need for improved pre-analytic procedures to allow molecular and genetic analysis.

Formalin has been the standard fixative for many decades, resulting in substantial archives of formalin-fixed and paraffin-embedded samples of BE and EAC in pathology departments. These well-defined repositories are frequently used for molecular cancer research. While (mi)RNA, DNA, and proteins can be isolated from FFPE samples, the cross-linking property of formaldehyde leads to poor-quality molecules and varying degrees of molecular degradation depending on fixation times [15–17]. Thus, alternative fixation solutions, which allow both high-quality molecular and histomorphological analyses, may have an advantage over FFPE when including molecular analyses as a diagnostic adjunct. The formalin-free PAXgene tissue preservation technology simultaneously preserves tissue morphology and antigenicity as well as nucleic acids, proteins, and phosphoproteins in tissue samples [18–25]. The PAXgene tissue system uses a non-cross-linking, non-carcinogenic combination of different alcohols, acids, and a soluble organic component to preserve both morphology and biomolecules.

As a prerequisite for the implementation of PAXgene in the clinical routine of BE diagnostics, the non-inferiority of tissue preservation using PAXgene compared to the current gold standard formalin still needs to be shown by independent and blinded trials. Thus, the aim of this study was to evaluate the quality of histo- and cytomorphological features and interobserver variability of PAXgene-fixed paraffin-embedded (PFPE) biopsies in comparison to routine formalin-fixed paraffin-embedded (FFPE) samples. In an international ring trial, nine experienced Barrett’s pathologists from Europe, the USA, and Australia blindly reviewed a balanced number of PFPE and FFPE biopsies from across the diagnostic spectrum (NDBE, LGD, HGD, and EAC). Using virtual microscopy and a standardized evaluation form, the study compares overall quality, different cyto- and histomorphological features, and diagnostic reproducibility between the PAXgene system and state-of-the-art FFPE technique for BE and EAC diagnostics.

## Materials and methods

### Assessors

Twenty-one international gastrointestinal (GI) pathologists were approached to join the study through direct professional contacts. Nine pathologists responded, were recruited to the study, and completed the case set of hematoxylin and eosin (H&E)– and PAS-stained slides (three pathologists from Germany, two from the USA, and one from Australia, France, the Czech Republic, and the Russian federation each). All participating pathologists had at least 5 years of professional experience in gastrointestinal pathology and a work setting of an academic teaching hospital. Participating pathologists received emails detailing the study objective and study design and were provided with personal log-in credentials to the online platform (Aperio eSlide Manager) and the online evaluation survey, described in the digital case assessment section. The lead study author (MB) provided assistance with participating pathologists’ log-in queries and evaluated the study progress. Pathologists of the Institute of Pathology of the Technical University of Munich (including two study authors WW and JSH) with experience in BE dysplasia assessment and the PAXgene tissue technology reviewed all digital slides.

### Case selection, scanning, and digital case assessments

The senior study author (JSH) selected a representative case mix of 116 BE biopsy cases from across the diagnostic spectrum. Inclusion criteria were diagnosis confirmed by a second GI pathologist (internal or external, including the study author WW) and tissue slides as well as tissue block available. All cases were treatment-naïve. The study case collection consisted of a total of 59 FFPE cases (15 NDBE, 15 LGD, 15 HGD, and 14 EAC) and 57 PFPE cases (14 NDBE, 15 LGD, 14 HGD, and 14 EAC). FFPE cases (from 2013 to 2017) and PFPE cases (from 2014 to 2017) were retrieved from the BarrettNET registry [26] enrolled at Klinikum rechts der Isar, Technical University of Munich. For FFPE processing, biopsies were fixed in 10% neutral-buffered formalin immediately after endoscopy. For PFPE processing, biopsies were fixed using the PAXgene Tissue Fix (PreAnalytiX GmbH, Hombrechtikon, Switzerland) according to the manufacturer’s protocol for 2–4 h and transferred into PAXgene tissue stabilizer to stop the fixation process. Using a standard protocol, PAXgene-treated and formalin-fixed tissues were dehydrated and embedded in paraffin. In the case of PAXgene, low-melting temperature paraffin was used. Sections from all 116 samples were stained with hematoxylin and eosin (H&E) and periodic acid-Schiff (PAS) for 111 samples. Slides were scanned at × 40 resolution, comparable to × 400 magnification of conventional light microscopy, using a NanoZoomer Digital Pathology (NDP) slide scanner (Hamamatsu, Japan). Scans were checked for focus and acuity and rescanned if necessary. Subsequently, slides were anonymized, randomized, and blinded for the fixation method and uploaded to the password-protected Aperio eSlide Manager (Leica Biosystems). A short user manual for the Aperio eSlide Manager and morphometric features was provided with the study protocol that was sent to each participating pathologist. Each participant was asked to evaluate all virtual cases on quality criteria, relevant features for BE/EAC diagnostics, and dysplasia grading and to give a final diagnosis according to the Vienna classification based on the provided H&E and PAS staining (more details regarding evaluated items in Table 1). Results were entered into an electronic evaluation survey for each eSlide (Lime survey).

**Table 1:** Evaluated items. Table shows evaluated items for each case with respective answer possibilities

Criteria	Answer possibilities
Quality criteria	
• Chromatin	Excellent, good, satisfactory, weak, poor
• Nucleoli	Excellent, good, satisfactory, weak, poor
• Mitosis	Excellent, good, satisfactory, weak, poor
• Basal lamina	Excellent, good, satisfactory, weak, poor
• Mucin or goblet cells	Excellent, good, satisfactory, weak, poor
• Retraction artifact	Significant, moderate, minor, not present
• Edge artifact	Significant, moderate, minor, not present
Overall quality	Excellent, good, satisfactory, weak, poor
BE/EAC-specific features	
• Atypical mitosis	Yes, no
• Abnormal shapes	Yes, no
• Border irregularities	Yes, no
• Pseudostratification	Yes, no
• Crowding	Yes, no
• Invasiveness	Yes, no
• Surface maturation	Maintained, lost
Final diagnosis	NDBE, LGD, HGD, IFD, EAC
Certainty of diagnosis	Sure, most probably, unsure

*NDBE*, non-dysplastic Barrett’s esophagus; *LGD*, low-grade dysplasia; *HGD*, high-grade dysplasia; *IFD*, indefinite for dysplasia; *EAC*, esophageal adenocarcinoma

### Ethical approval

The patient studies were conducted in accordance with the Declaration of Helsinki. The ethical committee of the Technical University of Munich approved the study. Written informed consent was obtained from all patients as part of the BarrettNET registry [26].

### Statistical analysis

Comparisons of tissue quality features, architectural changes, and cytological abnormalities between fixation methods were assessed by the chi-square tests. *p* values < 0.05 were considered significant.

The interobserver reproducibility was assessed by considering the histological diagnosis (NDBE, LGD, HGD, IFD, EAC) as the pivotal variable. The interobserver variability between participating pathologists was evaluated by computing Fleiss’ kappa statistics (*κ*_*f*_) together with the relative 95% confidence interval (CI) [27]. The values mostly vary between 0 (no agreement) and 1 (absolute agreement). A negative value may be obtained in situations where the actual agreement is less than a chance one. The magnitude of the agreement for each *κ*_*f*_ was interpreted on the basis of the Landis and Koch classification criteria [28]. All statistical analyses were performed in R (version 4.1.2).

## Results

### Study design

**Fig. 1:**
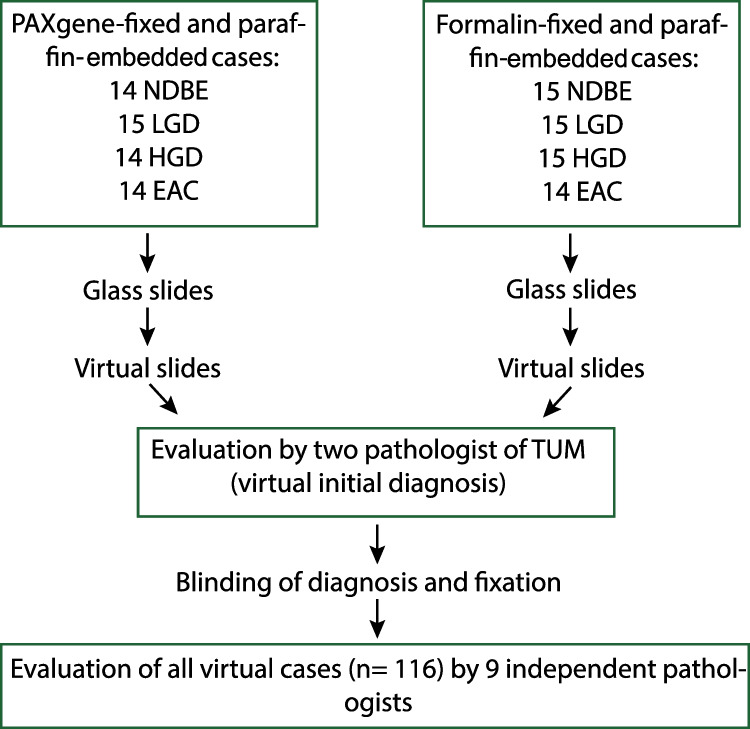
Study design. Similar numbers of NDBE, LGD, HGD, and EAC cases were either PAXgene-fixed or formalin-fixed and paraffin-embedded. The diagnosis and fixation method was blinded for each participant. Each participating pathologist (*n* = 9) was asked to evaluate all 116 virtual cases. NDBE, non-dysplastic Barrett’s esophagus; LGD, low-grade dysplasia; HGD, high-grade dysplasia; EAC, esophageal adenocarcinoma; TUM, Technical University Munich

Nine pathologists from six countries (Germany, France, the USA, Australia, Russia, Czech Republic) agreed to participate in the ring trial. Links to the randomized virtual slides and to the evaluation surveys were distributed to the participants together with the study protocol and detailed instructions. Each participant was asked to evaluate all 116 biopsies virtual cases, for which the diagnosis and fixation method was blinded. A total of 95 cases were evaluated by all nine participants, 20 cases by eight participants, and one case by seven participants. Each participant was asked to evaluate the quality of seven basic histo- and cytomorphological parameters and the quality of seven histomorphological features, relevant for BE/EAC diagnostics and dysplasia grading (see Table 1). Overall quality and a final diagnosis should be given for each case. In total, 7049 items were evaluated by the pathologists regarding histo- and cytomorphological quality parameters and 7154 items regarding relevant features for BE/EAC diagnostics and dysplasia grading. In total, 1022 overall quality scores and final diagnosis were assessed, respectively. The study design is illustrated in Fig. 1.

### Comparison of overall quality and histological and cytological features between PFPE and FFPE biopsies

**Fig. 2:**
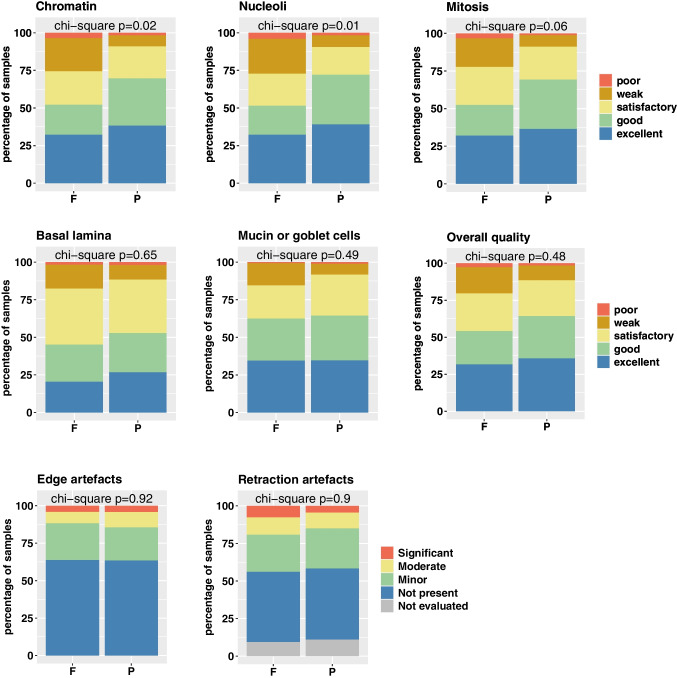
Comparison of quality parameters between PFPE and FFPE samples in Barrett’s esophagus and EAC diagnostics. Bar charts show different quality parameters for tissue architecture and cytological parameters for formalin-fixed paraffin-embedded (F) and PAXgene-fixed paraffin-embedded (P) samples. Chi-square tests were performed, and *p* values were annotated in the charts

**Fig. 3:**
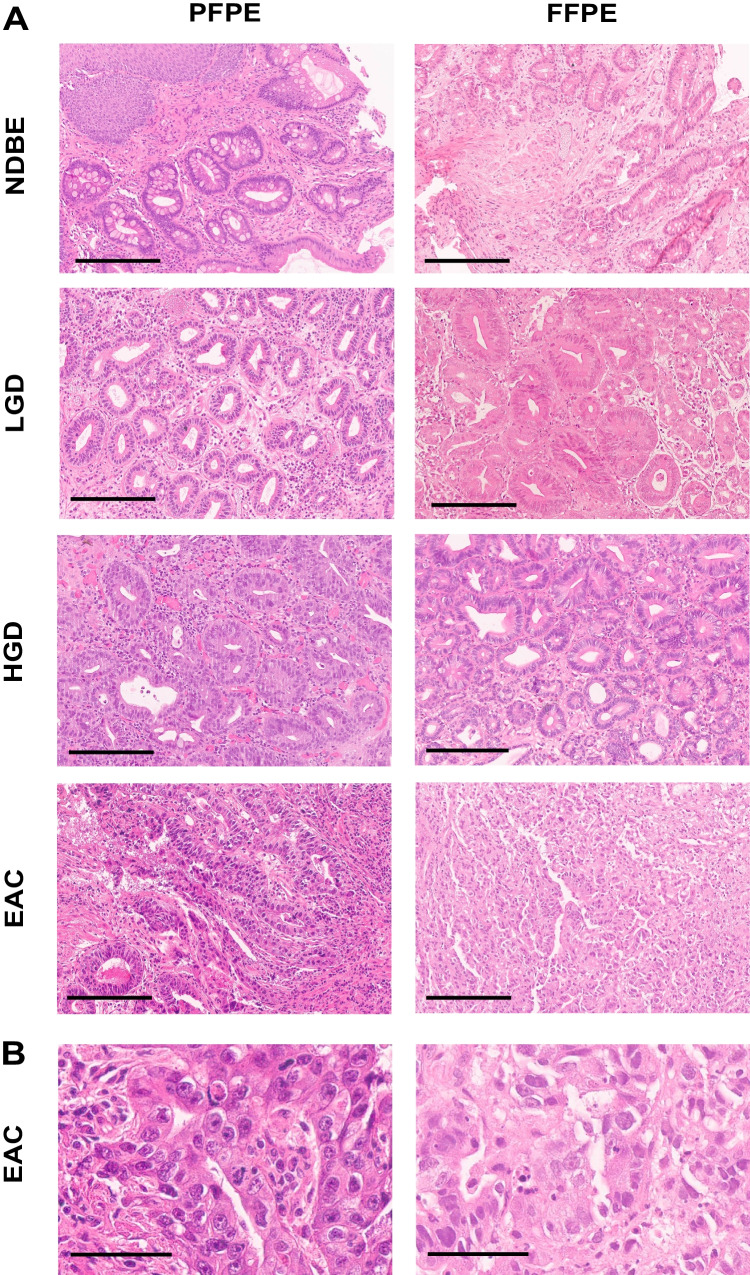
H&E staining of NDBE, LGD, HGD, and EAC tissue in PFPE and FFPE fixation. PFPE (left column) and FFPE tissue (right column) of **A** NDBE (top row), LGD (2nd row), HGD (3rd row), and EAC (bottom row). All scale bars represent 200 µm. **B** Higher magnification of EAC sample showing nuclear details. Scale bars represent 60 µm

A total of seven quality parameters for histological and nuclear features (quality of basal lamina, quality of mucin or goblet cells, retraction artifacts, edge artifacts and quality of chromatin, quality of nucleoli, quality of mitosis) and the overall quality were assessed by the participating pathologists in 116 cases. The resulting 7049 quality scores were separately analyzed in FFPE and PFPE samples. Nuclear quality was judged significantly superior in PFPE than in FFPE samples. In detail, the quality of chromatin and nucleoli was significantly better in PFPE samples than in FFPE samples (*p* = 0.02, *p* = 0.01), with over 65% of PFPE samples being classified as either excellent or good (Fig. 2), compared to ~ 50% of FFPE samples. Moreover, the quality of mitosis was judged better in PFPE than in FFPE, which did not reach the significance level though (*p* = 0.06). Exemplary slides in high magnification to show nuclear details for both fixation methods are shown in Fig. 3B. Further histological parameters evaluated such as the quality of basal lamina, mucus, and goblet cells and the overall quality were classified as either excellent or good in the majority of samples and did not differ between PFPE and FFPE (Fig. 2, *p* = 0.65, 0.49, and 0.48 respectively). Less than 5% of samples were judged as poor. The analysis of the parameters retraction and edge artifacts revealed no differences between PPFE and PFPE samples (Fig. 2, *p* = 0.92 and 0.9, respectively). In more than 75% of FFPE and PFPE samples, edge and retraction artifacts were not present or only minor. Figure 3A displays exemplary virtual slides used in the trial showing HE-stained PFPE and FFPE for non-dysplastic BE, LGD, HGD, and EAC samples, representing those cases with the best overlap of the diagnosis within the different pathologists.

### Comparison of relevant features for Barrett’s esophagus diagnostics and dysplasia grading between PFPE and FFPE

**Fig. 4:**
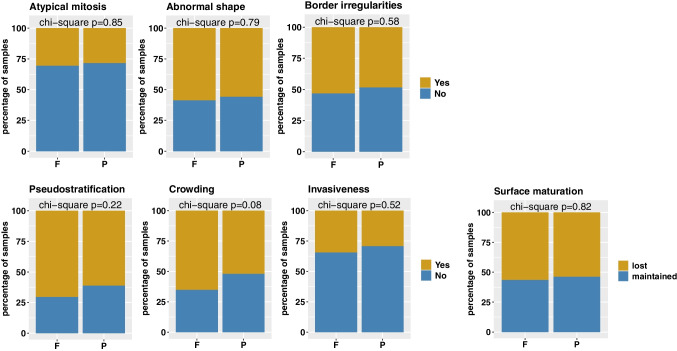
Comparison of specific features for BE and EAC diagnostics in PFPE and FFPE. Bar charts show the presence and absence of diagnostically relevant features for BE and EAC diagnostics in formalin-fixed paraffin-embedded (F) and PAXgene-fixed paraffin-embedded (P) samples. Chi-square tests were performed, and *p* values were annotated at the top of each chart

Next, we evaluated differences in the presence/absence of morphological features relevant for BE diagnostics and grading of dysplasia in FFPE and PFPE samples (Fig. 4). The presence of cytomorphological features important for the assessment and grading of dysplasia, such as atypical mitosis, abnormal nuclear shape, and nuclear membrane irregularities, did not differ between FFPE and PFPE samples (*p* = 0.85, *p* = 0.79, and *p* = 0.58). Moreover, the frequency of morphological features such as pseudostratification, gland crowding, invasiveness, and surface maturation (scores as present or absent) did not vary between FFPE and PFPE samples (*p* = 0.22, *p* = 0.08, *p* = 0.52, *p* = 0.82).

### Interrater reproducibility of diagnosis

**Fig. 5:**
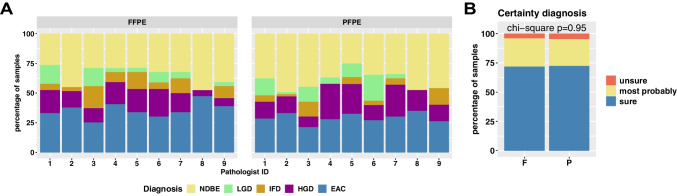
**A** Distribution of diagnoses of participating pathologists. Bar charts show distributions of diagnoses of each pathologist for FFPE samples (left) and PFPE samples (right). NDBE, non-dysplastic Barrett’s esophagus; LGD, low-grade dysplasia; HGD, high-grade dysplasia; EAC, esophageal adenocarcinoma. **B** Subjective certainty of pathologists’ diagnoses. Bar charts show a level of subjective certainty in pathologists’ diagnoses for formalin-fixed paraffin-embedded (F) and PAXgene-fixed paraffin-embedded (P) samples. Chi-square tests were performed, and *p* values were annotated in the charts

A final diagnosis was given according to the Vienna classification criteria [2] for each case, resulting in a total of 1022 diagnoses: 378 NDBE (37%), 71 LGD (7%), 168 HGD (16%), 333 EAC (33%), and 72 IFD (7%). We assessed the frequency of diagnosis given by individual pathologists in FFPE and PFPE samples (Fig. 5), which were not significantly different between fixation methods for most pathologists (chi-square test *p* > 0.05). Exceptions were pathologists 4 and 8, who showed significant differences in the frequencies of diagnoses between the FFPE and PFPE sample cohort (chi-square test *p* = 0.005 and *p* = 0.01). These two pathologists showed differences mainly in the categories IFD, LGD, and HGD, which are the categories with known high interobserver variability. Of note, the exclusion of these evaluations did not increase interobserver reliability neither for PFPE samples nor for FFPE samples (data not shown). Overall, we observed a decrease in the frequency of IFD diagnosis in PFPE compared to FFPE with a mean of 9.8% and 6.1% of samples being diagnosed as IFD in PFPE and FFPE, respectively, which was not significant though (*t*-test *p* value = 0.2). Concerning the parameter “How sure are you about the diagnosis?,” all participating pathologists felt equally confident in their diagnosis when evaluating PFPE or FFPE samples and were “sure” in their diagnosis in ~ 75% of the samples (Fig. 5).

Next, we assessed interrater reliability for individual diagnoses and fixation methods. The overall level of agreement on the entire sample cohort (FFPE and PFPE biopsies) was moderate, with a *κ*_*f*_ value of 0.54 (CI 0.52–0.56). As expected, the level of agreement was highest for NDBE and EAC (*κ*_*f*_ = 0.72 and *κ*_*f*_ = 0.75, respectively) and poorest for LGD (*κ*_*f*_ = 0.15). Next, we analyzed interrater agreement separately for PFPE and FFPE samples (Table 2). Regarding all cases included, interobserver variability was not remarkably different between FFPE and PFPE samples (PFPE *κ*_*f*_ = 0.53; FFPE: *κ*_*f*_ = 0.54). Kappa scores were marginally higher for HGD and EAC in PFPE samples (PFPE: *κ*_*f*(HGD)_ = 0.3, *κ*_*f*(EAC)_ = 0.75; FFPE: *κ*_*f*(HGD)_ = 0.24, *κ*_*f*(EAC)_ = 0.74), while slightly higher values were noted for LGD in FFPE samples (PFPE: *κ*_*f*(LGD)_ = 0.13, FFPE: *κ*_*f*(LGD)_ = 0.18). When combining the diagnoses of HGD and LGD to a common category of “any dysplasia,” better interrater reliability was achieved with marginally higher kappa scores for PFPE than for FFPE (PFPE: *κ*_*f*(dysplasia)_ = 0.49, FFPE: *κ*_*f*(dysplasia)_ = 0.42). We further evaluated kappa scores in a combined category for HGD and EAC and observed a substantial improvement of interrater reliability in both PFPE and FFPE (PFPE: *κ*_*f*(HGD/EAC)_ = 0.63, FFPE: *κ*_*f*(HGD/EAC)_ = 0.75).

**Table 2:** Interrater variability: The table shows kappa Fleiss scores for the entire study cohort including all diagnostic categories

Categories	PFPE	FFPE
All cases (*n* = 116)	0.53	0.54
NDBE	0.72	0.72
EAC	0.75	0.74
HGD	0.30	0.24
LGD	0.13	0.18
Any dysplasia	0.49	0.42
HGD or EAC	0.63	0.75

*NDBE*, non-dysplastic Barrett’s esophagus; *EAC*, esophageal adenocarcinoma; *HGD*, high-grade dysplasia; *LGD*, low-grade dysplasia

## Discussion

The Vienna classification remains the most commonly used predictor of esophageal adenocarcinoma risk. As it is based on morphological and cytological characteristics, high histomorphology quality is required for subsequent risk stratification and treatment decision [1–3]. However, due to the poor interobserver reproducibility of Barrett’s esophagus diagnostics, novel strategies for progression risk prediction are in demand, including molecular/genetic biomarkers and artificial intelligence. The formalin-free PAXgene system, as an alternative tissue preservation method to the standard formalin fixation, enables histopathological analysis and preserves biological molecules at the highest quality, which could improve multimodal analytics. Thus, we performed an international ring trial to evaluate the quality of histo- and cytomorphological features of PAXgene-fixed, paraffin-embedded (PFPE) biopsies and their suitability for histomorphological Barrett’s esophagus diagnostics in comparison to the gold standard formalin-fixation and paraffin-embedding (FFPE). Nine gastrointestinal pathologists from six countries and three continents blindly evaluated digitized slides from 57 PFPE and 59 FFPE biopsies from across the diagnostic spectrum of Barrett’s esophagus. Overall quality, the quality of cytological and histomorphological features, and the presence of artifacts were scored, and a diagnosis was given for each biopsy.

The evaluation of in total 7049 quality scores revealed that the quality of histo- and cytomorphological features was judged as equal or even better in PFPE compared to FFPE. The quality of nuclear features was perceived as being of significantly better quality in PFPE than in FFPE. Features such as chromatin and nucleoli were scored significantly better in PFPE than in FFPE. As already described in previous ring trials for colorectal cancer [25] and non-neoplastic tissue of different organs [21], both hematoxylin and eosin (H&E) and periodic acid-Schiff (PAS) staining were more intense in PPFE than FFPE samples, resulting in a stronger contrast. This might explain the perception of a higher nuclear quality of PFPE samples, as details such as chromatin, nucleoli, and mitosis are easy to recognize. Nevertheless, this did not translate to improved interobserver agreement in our study cohort. The classification of Barrett’s dysplasia follows international standards, such as the Vienna classification. It is based on different degrees of morphological and cytological changes and assigns those to different grades of dysplasia. Even though such a classification leads to better agreement between different pathologists, the interobserver agreement is still unsatisfactory. This is due to the fact that morphological changes are classified in semi-quantitative degrees (very subtle, mild, severe), and the integration of those semi-quantitative changes results in a final classification or diagnosis and presupposes a weighting of the individual changes. It is therefore not surprising that even among international experts, despite the application of the same standards, an individual scope of interpretation leads to a different diagnostic evaluation, especially in the category of low-grade dysplasia and in the use of the category indefinite for dysplasia (best seen for pathologists 4 and 8).

Previous studies showed that the histomorphology of tissues fixed in PAXgene fixative for only 3 h was comparable to that of tissues fixed in formalin for 6–8 h, indicating the potential in improving laboratory workflow through time shortening from sample acquisition to diagnosis in clinical routine pathology [21]. Despite tremendous efforts in the screening of BE patients, the time-effectiveness and cost-effectiveness of current surveillance strategies in reducing EAC mortality are debatable [29, 30] as > 90% of patients presenting with EAC have no prior diagnosis of BE [29, 31], and the majority of patients with BE enrolled in surveillance programs will never progress to cancer [32, 33]. One reason is the challenge of the high variability of dysplasia grading among pathologists [4–6]. In this ring trial of 116 study cases equally balanced concerning diagnostic categories (NDBE, LGD, HGD, EAC), most pathologists showed no significant difference in their diagnosis frequencies between the cohort of PFPE and FFPE samples. Comparing the interobserver variability, we observed only moderate interrater reproducibility in the overall cohort; however, there was no significant difference between PFPE and FFPE. Not surprisingly, there was substantial agreement between pathologists for the diagnosis of NDBE and EAC and low agreement in LGD and HGD, both in FFPE and PFPE. Thus, the results of this ring trial confirm previous studies reporting a high interobserver variability in dysplasia grading, highest among LGD (*κ* = 0.11–0.3) [4–6].

One strategy to reduce interobserver variability would be the integration of molecular or genetic markers. p53 IHC in addition to standard H&E staining has been shown to significantly increase interobserver agreement [34], and abnormal p53 IHC has been shown to correlate with a risk of progression [9]. p53 IHC showed equal immune reactivity on PFPE sections compared to FFPE sections, if antigen retrieval was performed [35]. Moreover, the integration of artificial intelligence systems is based on deep learning to support pathologist grading. Artificial intelligence (AI) systems have been shown to significantly improve the grading of other (pre)malignant entities [36–39]. Our study showed significantly better preserved nuclear features such as chromatin and nucleoli together with the increased contrast in PFPE samples, which could potentially increase the amount of valuable information for AI systems for higher performance in image analysis and risk prediction. This could potentially impact diagnostic accuracy, as it was shown by digital analysis, that chromatin texture was the best discriminator for the diagnosis of Barrett’s dysplasia [40].

Over the last decades, molecular analyses have been implemented to discriminate patient groups for risk stratification and treatment decision to allow personalized molecular prevention. Recent studies in the field of BE showed that DNA-derived markers such as the detection of changes in copy number patterns and chromosomal instability are promising biomarkers [10–12]. Some studies suggest that the risk of BE progression can be predicted by the degree of clonal diversity [13, 41]. Although this study focuses on the histomorphological aspects of PAXgene tissue fixation, investigations of previous PAXgene studies have already investigated the quality of nucleic acids and proteins derived from PFPE tissues. These studies verified that the formalin-free PAXgene fixation technology preserves DNA similar to DNA derived from snap-frozen tissue [24, 42, 43]. Moreover, RNA quality and reliability of gene expression data are similar in PFPE and snap-frozen samples [23], whereas gene expression profiling from FFPE tissue–derived RNA is affected by a high level of fragmentation [24, 44]. In proteomic testing, proteins derived from PFPE tissue showed a reactivity pattern analogous to proteins derived from frozen tissue, whereas proteins isolated from FFPE tissue showed reduced activity [18]. Therefore, the formalin-free PAXgene method offers the opportunity for both high-quality analysis of a broad spectrum of biomolecules along with histomorphological analysis from the same tissue sample.

This international ring trial performed by nine experienced GI pathologists evaluated the suitability of the formalin-free PAXgene tissue system for BE diagnostics. Our results show that histomorphological tissue quality is better preserved in BE biopsies using the PAXgene tissue stabilization system, as the quality was judged equal or even better in PFPE than in FFPE biopsies. With a rising spectrum of diagnostic tools in medical care such as genetic, epigenetic, and proteomic techniques and AI image analysis for improvement of diagnosis standardization and treatment decisions, the non-cross-linking formalin-free PAXgene fixation could therefore substantially contribute to the advancement in molecular-guided BE and EAC diagnostics and surveillance.
